# Evaluation of serum MUC5AC in combination with CA19-9 for the diagnosis of pancreatic cancer

**DOI:** 10.1186/s12957-020-1809-z

**Published:** 2020-02-07

**Authors:** Jiayu Zhang, Yue Wang, Tiancheng Zhao, Yezhou Li, Leilei Tian, Jinming Zhao, Jingxin Zhang

**Affiliations:** 1 Department of Gastrointestinal and Colorectal Surgery, China-Japan Union Hospital of Jilin University, Changchun, China; 2 Department of Hepatology, The Fifth People’s Hospital of Suzhou, Suzhou, China; 3 Department of Endoscopy Center, China-Japan Union Hospital of Jilin University, Changchun, China; 4 Department of Vascular Surgery, China-Japan Union Hospital of Jilin University, Changchun, China; 5 Operating Room, China-Japan Union Hospital of Jilin University, Changchun, China; 6 Department of General Surgery, Affiliated People’s Hospital of Jiangsu University, Zhenjiang, China

**Keywords:** Biomarker, Carbohydrate antigen 19-9 (CA19-9), Mucin 5AC (MUC5AC), Pancreatic cancer (PC)

## Abstract

**Background:**

Pancreatic cancer (PC) is a highly aggressive tumor with a poor prognosis that lacks specific diagnostic markers. Mucin 5AC (MUC5AC) is a member of the mucin family, a heterogeneous group of high molecular weight, heavily glycosylated proteins that could be either membrane-bound or secreted. This multi-central study is to evaluate the performance of serum MUC5AC in combination with carbohydrate antigen 19-9 (CA19-9) for the diagnosis of PC in Asian.

**Methods:**

Sixty-one patients with PC (comprised of early pancreatic cancer [*n* = 30] and late pancreatic cancer [*n* = 31] patients), 29 benign control, 35 choledocholithiasis, 25 chronic pancreatitis, and 34 healthy controls, were recruited from two hospitals. Serum levels of MUC5AC were evaluated by commercial ELISA kits. CA19-9 was measured by chemiluminescence immunoassay. The cutoff value of MUC5AC was determined based on optimal sensitivity and specificity.

**Results:**

Serum MUC5AC in patients with PC (210.1 [100.5–423.8] ng/mL) presented higher levels than those in controls. The combined biomarker panel (MUC5AC and CA19-9) presented better performance and improved specificity to differentiate PC from controls (AUC 0.894; 95% CI (0.844–0.943), sensitivity 0.738, specificity 0.886) than CA19-9 (*p* = 0.043) or MUC5AC alone (*p* = 0.010); however, the latter two had no difference (*p* = 0.824).

**Conclusions:**

Serum MUC5AC is a potential biomarker for PC. The combination with CA19-9 presents improved specificity and better performance.

## Introduction

Pancreatic cancer (PC) is a highly aggressive tumor with a poor prognosis [1]. The past decades witness the constant increase of the incidence, and it is going to become the second most fatal cancer in 2030 [2].

In the clinic, there are still no reliable early detection tools and limited options of therapy, which lead to a dismal prognosis [1, 3]. Thus, it is urgent to identify potential biomarkers for PC detection [4]. At present, the only widely used biomarker in the routine management of PC is CA19-9, an epitope of sialylated Lewis blood group antigen [5]. It has a fine diagnostic performance, with a sensitivity of 80% in symptomatic patients; however, it also increases in pancreatic inflammation and benign pancreatic disease, which hampers its specificity [6]. Thus, large numbers of studies are being performed to identify an accurate tumor marker that could promote PC management; however, few hold promise as a preferred biomarker [7]. To improve the specificity of identifying PC, a panel that consists of multiple biomarkers might be a good solution [8, 9].

Mucin 5AC (MUC5AC) is a member of the mucin family, a heterogeneous group of high molecular weight, heavily glycosylated proteins that could be either membrane-bound or secreted [10]. Recently, Kaur et al. [11] evaluated the utility of MUC5AC in PC detection at the levels of tissue and circulating, in a multi-central Caucasian population. Serum levels of MUC5AC were measured by the sandwich ELISA developed in-house (anti-MUC5AC antibody # ab77576, Abcam, Cambridge, MA). They found its circulating levels in patients with PC, either in early-stage or late-stage, were higher in comparison with benign hepatobiliary/pancreatic diseases and chronic pancreatitis (CP). It also suggested an acceptable performance of MUC5AC in differentiating resectable early-stage PC from controls. Moreover, a combination of MUC5AC and CA19-9 significantly improved the accuracy for identifying resectable early-stage PC.

Biomarker candidates tend to present promising potential; unfortunately, their applications are found to be limited, largely because of the diversities in ethnicity, epidemiological background, lifestyle, diet, analytical methods, etc. Previous studies confirm the racial and ethnic disparities in PC, e.g., incidence, histologic types, and survival [12, 13]. The explanations for the differences include biological disparities, environmental factors, and tumor characteristics [14]. A meta-analysis by Pei et al. [15] suggests significant divergences between Caucasian and Asian subgroups for circulating miRNA profiles. Given this background, the establishment of standard test and multi-center/ethnicity validation is essential to reduce the variability [16]. Blood sample is minimally invasive to obtain and is most widely used in laboratory tests [4, 5]. Given the frustrated situation that single biomarkers are unlikely to provide reliable accuracy, researchers have been working on the qualified panels of biomarkers for years [17]. Here, we aim to evaluate the performance of serum MUC5AC in combination with CA19-9 for the diagnosis of PC in a multi-central Asian population.

## Methods

### Study design

The study is one part of a longitudinal study *Clinical Application Of Serum Biomarkers In Digestive Cancers*. Clinical data and blood sample were collected from two hospitals, i.e., Affiliated People’s Hospital of Jiangsu University and China-Japan Union Hospital of Jilin University, from June 2013 to December 2017. Sixty-one patients with PC (comprised of early pancreatic cancer [*n* = 30] and late pancreatic cancer [*n* = 31] patients), 29 benign control (BC), 35 choledocholithiasis (CDL), 25 CP, and 34 healthy controls (HC), were recruited in this study. Written informed consent was obtained from each participant. This study was approved by the Ethics Committee of two hospitals (Ethical approval number 2011CJUH-ER-015 and 2012JSU-ER-003), in accordance with the Helsinki Declaration of 1975.

### Inclusion criteria

PC was limited to pancreatic adenocarcinoma. The diagnosis and staging were determined surgically, based on operative pathology or biopsy of metastatic disease [1, 18]. Therapy included surgery and chemotherapy. The samples prior to surgery or the initiation of systemic chemotherapy were classified as pre-therapy.

BC: Patients with benign pathologies, e.g., pancreatic pseudocysts and serous cystadenomas, were categorized as BC [19].

CDL and CP were diagnosed based on the standard clinical practices [20, 21].

HC: the inclusion criteria were the following: (1) normal liver biochemistry, (2) no history of hepatobiliary or pancreatic disease, (3) no other systematic diseases, and (4) no malignant disease.

### Laboratory examination

Serum levels of MUC5AC were evaluated by commercial ELISA kits (catalog no. CSB-E10109h; CUSABIO CORP., China). In this evaluation, the intra- and inter-assay CVs of MUC5AC test were 4.7% and 11.5%, individually. Serum levels of CA19-9 were measured by chemiluminescence immunoassay with an Abbott-Architect immunoanalyzer (Abbott Laboratories, Abbott Park, IL).

### Statistical analysis

Differences among various groups (more than two) used the Kruskal–Wallis test. Receiver operating characteristics (ROC) curves were developed to evaluate sensitivity, specificity, and areas under the curves (AUCs) with 95% CI. The cutoff value of MUC5AC was determined based on optimal sensitivity and specificity. To evaluate whether the combination of markers was better than either alone, a new variable predicted probability (pp) for PC was developed based on an equation obtained by binary logistic regression. The independent variables include serum levels of MUC5AC and CA19-9, while the dependent variable is the binary outcome, i.e., PC vs. all controls. Statistics were analyzed using SPSS (version 24.0, SPSS Inc., Chicago, IL, USA) and Stata (version MP 11.2, StataCorp LP, College Station, TX, USA). The power of the sample size was calculated by G*Power (version 3.1, Heinrich-Heine-Universität Düsseldorf, Germany) [22]. A two-sided *p* < 0.05 was considered statistically significant.

## Results

The characteristics of all participants were indicated in Table 1.

**Table 1:** Characteristics of participants

	Healthy controls	Benign controls	Choledocholithiasis	Chronic pancreatitis	Pancreatic cancer (*n* = 61)	
					Early pancreatic cancer	Late pancreatic cancer
Number	34	29	35	25	30	31
Sex						
Male	23	18	19	21	16	17
Female	11	11	16	4	14	14
Age (years)	52.3 ± 11.3	55.1 ± 10.2	51.0 ± 10.2	52.1 ± 13.8	50.3 ± 10.4	52.0 ± 11.2
BMI (kg/m^2^)	24.0 ± 3.2	25.5 ± 4.2	24.8 ± 2.8	22.2 ± 4.0	22.7 ± 3.2	22.1 ± 3.4
ALT (U/L)	21 (14–27)	18 (13–28)	42 (28–57)	32 (20–76)	73 (50–102)	65 (42–131)
AST (U/L)	20 (18–22)	18 (15–26)	35 (27–52)	34 (26–53)	89 (67–106)	87 (70–100)
GGT (U/L)	23 (16–41)	34 (18–54)	60 (37–126)	45 (28–84)	134 (89–188)	145 (91–182)
TBIL (μmol/L)	14.4 (10.8–20.8)	16.0 (12.8–21.8)	32.0 (28.8–41.6)	16.0 (13.6–21.6)	58.8 (55.6–66.8)	62.0 (58.8–160.4)
DBIL (μmol/L)	6.4 (4.8–8.0)	6.4 (5.6–8.8)	18.4 (16.8–21.6)	6.4 (4.8–8.0)	46.8 (38.7–59.0)	48.4 (46.8–146.8)
Albumin (g/L)	44.2 ± 4.3	41.7 ± 4.0	43.1 ± 5.1	32.7 ± 4.5	32.2 ± 4.3	31.1 ± 3.6
CA19-9 (U/mL)	15.1 (8.4–25.2)	11.6 (8.8–34.6)	48.5 (27.6–61.4)	35.6 (27.4–44.7)	376.1 (83.1–552.7)	399.9 (48.5–687.3)
MUC5AC (ng/mL)	60.8 (37.8–81.3)	86.4 (48.4–108.2)	91.8 (68.0–127.4)	95.1 (59.7–160.3)	174.6 (87.3–377.0)	228.7 (105.5–596.3)

Data are mean ± standard deviation or median (interquartile range) for continuous variables

*ALT* alanine transaminase, *AST* aspartate aminotransferase, *BMI* body mass index, *DBIL* direct bilirubin, *GGT* γ-glutamyltransferase, *TBIL* total bilirubin

### Biomarkers levels

**Fig. 1:**
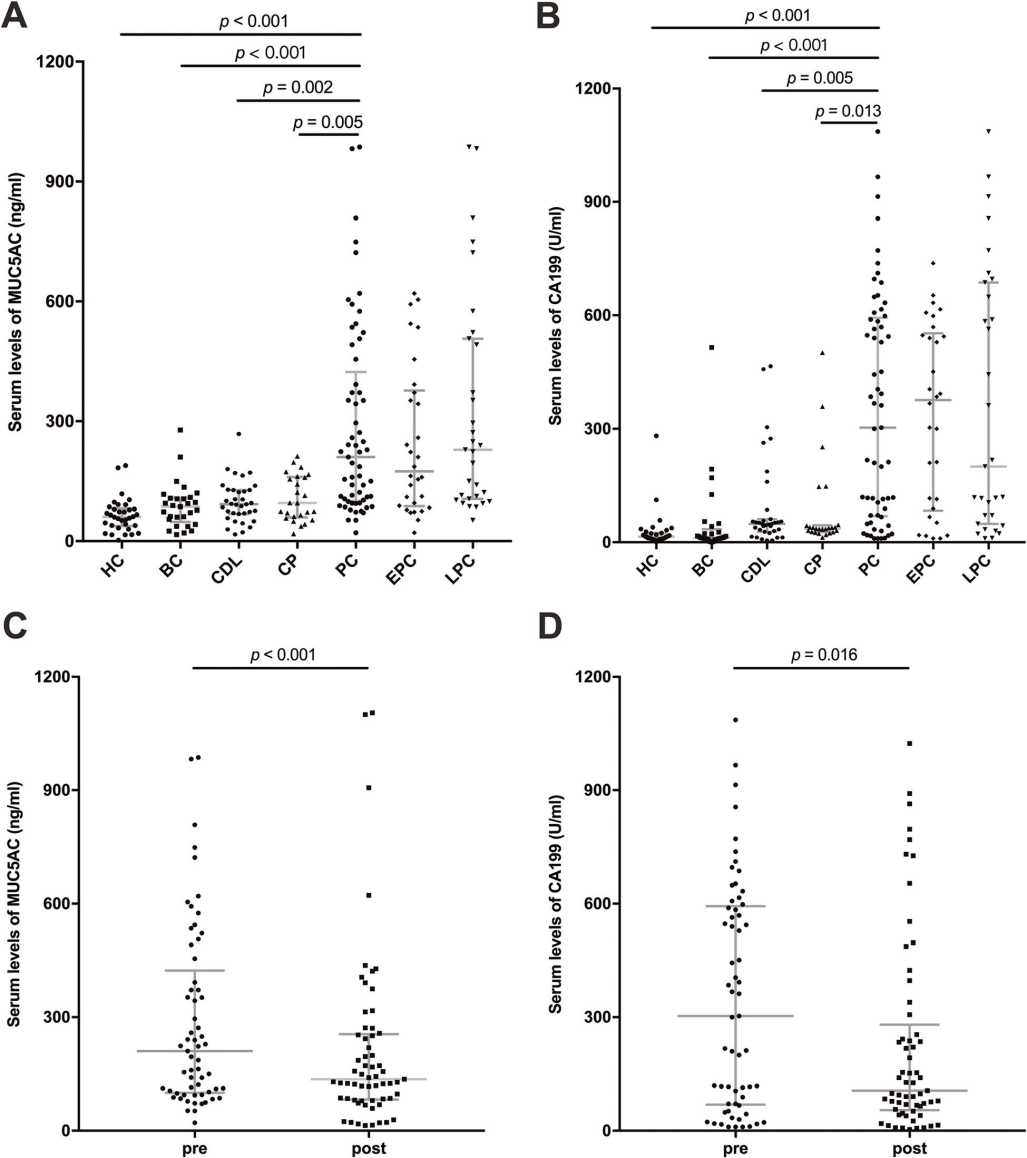
Serum levels of MUC5AC and CA19-9. **a** Levels of serum MUC5AC (ng/ml, median [interquartile range]). **b** Levels of serum CA19-9 (U/ml, median [interquartile range]). **c** Comparison of serum MUC5AC levels between pre- and post-therapy PC samples (ng/ml, median [interquartile range]). **d** Comparison of serum CA19-9 levels between pre- and post-therapy PC samples (U/ml, median [interquartile range]). PC, pancreatic cancer; therapy includes surgery and chemotherapy

Figure 1a showed that serum MUC5AC in patients with PC (210.1 [100.5–423.8] ng/mL) presented higher levels than those in controls (HC, 60.8 [37.8–81.3] ng/mL; BC, 86.4 [48.4–108.2] ng/mL; CDL, 91.8 [68.0–127.4] ng/mL; CP, 95.1 [59.7–160.3] ng/mL). Serum levels of CA19-9 were higher in patients with PC (303.1 [69.2–593.5] U/mL) than in all controls (HC, 15.1 [8.4–25.2] U/mL; BC, 11.6 [8.8–34.6] U/mL; CDL, 48.5 [27.6–61.4] U/mL; CP, 35.6 [27.4–44.7] U/mL), as shown in Fig. 1b.

The levels of the two markers both presented a significant difference between pre- and post-therapy samples. Post-therapy levels (136.1 [82.5–255.3] ng/mL) of MUC5AC significantly decreased from pre-therapy levels (210.1 [100.5–423.8] ng/mL, *p* < 0.001), in Fig. 1c. Similarly, Fig. 1d showed serum CA19-9 decreased from pre-therapy levels (303.1 [69.2–593.5] U/mL) to post-therapy levels (106.0 [54.2–280.3] U/mL, *p* = 0.016).

### Cutoff values and AUCs

**Fig. 2:**
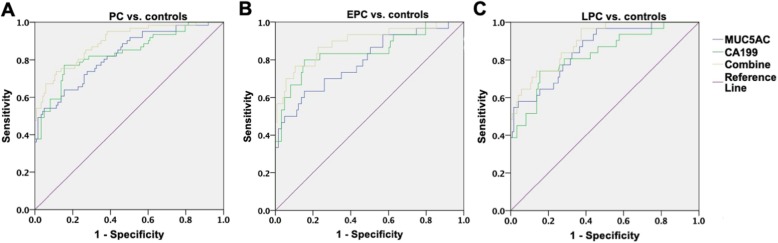
ROC curves for MUC5AC, CA199, and the combination in the diagnosis of PC. **a** ROC curves for MUC5AC, CA19-9, and the combination in PC vs. controls. **b** ROC curves for MUC5AC, CA19-9, and the combination in EPC vs. controls. **c** ROC curves for MUC5AC, CA19-9, and the combination in LPC vs. controls. EPC, early-stage pancreatic cancer; LPC, late-stage pancreatic cancer; PC, pancreatic cancer; ROC, receiver operating characteristic

In this study, we chose 37 U/mL as the cutoff value for CA19-9, based on the guideline [1, 6]. The AUC for PC was 0.836 (95% CI 0.770–0.902), with a sensitivity of 0.820 and specificity of 0.618 (Table 2 and Fig. 2a). ROC curves showed the optimal cutoff for MUC5AC was 185.6 ng/mL (AUC 0.825 [0.760–0.890], sensitivity 0.541, specificity 0.951).

**Table 2:** Diagnostic performance

	AUC	95% CI	Sensitivity	Specificity	PPV	NPV	Accuracy
MUC5AC							
PC vs. controls	0.825	0.760–0.890	0.541	0.951	0.846	0.807	0.815
EPC vs. controls	0.793	0.695–0.890	0.500	0.951	0.714	0.886	0.863
LPC vs. controls	0.856	0.783–0.930	0.581	0.951	0.75	0.900	0.877
CA19-9							
PC vs. controls	0.836	0.770–0.902	0.820	0.618	0.515	0.874	0.685
EPC vs. controls	0.844	0.752–0.937	0.833	0.618	0.347	0.938	0.660
LPC vs. controls	0.827	0.740–0.914	0.806	0.618	0.347	0.927	0.656
Combination							
PC vs. controls	0.894	0.844–0.943	0.738	0.886	0.763	0.872	0.837
EPC vs. controls	0.892	0.819–0.966	0.767	0.886	0.622	0.940	0.863
LPC vs. controls	0.895	0.837–0.954	0.710	0.886	0.611	0.924	0.851

*AUC* area under receiver operating characteristic curve, *EPC* early-stage pancreatic cancer, *LPC* late-stage pancreatic cancer, *NPV* negative predictive value, *PC* pancreatic cancer, *PPV* positive predictive value

### Combination

To investigate the diagnostic value of the combined markers, a new variable predicted probability (pp) for PC was developed based on the equation obtained by the binary logistic regression (PC vs. all controls). The equation was as follows: $\mathit{\ln}\left(\frac{\mathrm{pp}}{1-\mathrm{pp}}\right)=0.006\times \mathrm{CA}199+0.015\times \mathrm{MUC}5\mathrm{AC}-3.547$. In Fig. 2a, the optimal cutoff value for the new variable was 0.329. It indicated better identifying ability (AUC 0.894 [0.844–0.943], sensitivity 0.738, specificity 0.886), in comparison with MUC5AC (*p* = 0.010) and CA19-9 (*p* = 0.043), even though the latter two had no difference (*p* = 0.824).

### Early-stage PC (EPC) and late-stage PC (LPC)

In identifying EPC from the controls, the combination presented an improved AUC (0.892 [0.819–0.966]), while CA19-9 was 0.844 ([0.752–0.937], *p* = 0.299) and MUC5AC was 0.793 ([0.695–0.890], *p* = 0.012), in Fig. 2b and Table 2. The latter two still indicated no difference (*p* = 0.461). As far as LPC, the combination (0.892 [0.819–0.966]) performed better than CA19-9 (0.827 [0.740–0.914], *p* = 0.030), but not MUC5AC (0.856 [0.782–0.930], *p* = 0.225).

## Discussion

Minimally invasive, early diagnostic tools featured with high accuracy are desirable [8]. CA19-9, as a blood-based marker, shows only modest performance for PC diagnosis, with variable sensitivity and specificity owing to Lewis phenotype [1, 4, 23]. The previous studies suggest its combination with other markers may improve accuracy. In this study, MUC5AC shows a high identifying performance with good specificity, when combining with CA19-9, the sensitivity remarkably increase.

Previously, Iacobuzio-Donahue et al. [24] performed a comparative genomic analysis of normal pancreatic tissue and tissues of CP and PC. In the study, MUC5AC was firstly identified as a significantly higher expression of mucin gene in PC tissue, in comparison with benign pathologies. Furthermore, Kato et al. [25] found in the development of PC that Sp1 involves in MUC5AC promoter activity under basal conditions, while AP-1 involves in both basal and phorbol 12-myrisate 13-acetate-induced MUC5AC promoter activity in PC cells. The pathways of SP-1, PKC/ERK/AP-1, and PKC/JNK/AP-1 are essential in the regulation of MUC5AC transcription. Ohuchida et al. [26] showed that the transcript levels of MUC5AC were higher in pancreatic tumoral tissues than in non-tumoral tissues. Besides, MUC5AC mRNA in pancreatic juice presented fine diagnostic performance to identify PC. The study indicated a stepwise upregulation of MUC5AC from high-grade pancreatic intraepithelial neoplasia to invasive ductal carcinomas. Yamazoe et al. [27] found MUC5AC expression might be associated with the invasive progression of pancreatic ductal carcinoma, suggesting the role of MUC5AC in the acceleration of PC progression. An in vivo xenograft study by Hoshi et al. [28] showed that MUC5AC knockdown drastically downregulated the tumorigenicity and suppressed the tumor growth. MUC5AC inhibits the antitumor effect of neutrophils and neutrophils-induced apoptosis [29]. It supports the MUC5AC functions as an immunosuppressive agent and plays a key role in the escape of carcinoma cells from immunosurveillance. Sierzega et al. [30] evaluated mucin expression by immunohistochemistry in specimens from PC, CP, and normal pancreas. They developed a three-MUC diagnostic model, including MUC3, MUC5AC, and MUC6, which presented the potential to differentiate PC from non-malignancy. Similarly, Wiktorowicz et al. [31] examined mucin expression by PCR and immunohistochemistry in surgical and biopsy specimens from patients with pancreatic, ampullary, common bile duct cancers, and CP. It showed the panel of mucin expression profiling might be valuable in differentiating malignant lesions from PC. As an inspiration of previous findings, Yamashita et al. [32] recently reported a case in which immunohistochemical staining for MUC5AC helped in distinguishing PC from breast cancer metastasis, using the specimens obtained by endoscopic ultrasound fine-needle aspiration biopsy.

Given its secretory nature, MUC5AC holds promise as a potential diagnostic biomarker. In the USA, Kaur et al. [11] explored the diagnostic potential of MUC5AC alone and in combination with CA19-9 in a Caucasian population. In this study, we evaluate if the combination of MUC5AC and CA19-9 could improve the diagnostic accuracy for PC in Asian. This study follows the widely used protocol for the evaluation of diagnostic biomarkers [33]. It indicates that circulating levels of MUC5AC are significantly higher in patients with PC, compared with the controls, including HC, BC, CDL, and CP. The panel presents better performance than MUC5AC or CA19-9 alone to differentiate PC from non-malignancy. Given the fine sensitivity of CA19-9, the panel shows significantly improved specificity, even in identifying EPC. Furthermore, besides for early diagnosis, MUC5AC presents the potential to monitor patients’ response to clinical therapy. Further studies are required to evaluate the performance of MUC5AC in combination with CA19-9 in a cohort of patients with PC undergoing chemotherapy, radiotherapy, or surgical interventions. Lastly, Kaur et al. [11] developed in-house sandwich ELISA, whereas we use commercial ELISA kits, which suggest the practicability of MUC5AC commercialized test in PC diagnosis.

Our study has some limitations. Firstly, patients with other digestive carcinomas, e.g., hepatocellular carcinoma or cholangiocarcinoma, should be included to rule out possible false-positive results. Moreover, MUC5AC levels in subjects with certain pancreatic cysts that have malignant potential, e.g., mucinous cystic neoplasm and intraductal papillary mucinous neoplasm, require further investigation.

## Conclusions

This study is to evaluate the combination of circulating MUC5AC and CA19-9 for PC detection in Asian. The results suggest that MUC5AC is a potential biomarker for the clinical management of PC. Furthermore, the combination of the two tumor markers could significantly improve the accuracy and specificity in differentiating PC from benign controls, even early-stage PC.

## Data Availability

The datasets used and/or analyzed during the current study are available from the corresponding author on reasonable request.

## Abbreviations

AUC: Areas under the curve
BC: Benign controls
CA19-9: Carbohydrate antigen 19-9
CDL: Choledocholithiasis
CP: Chronic pancreatitis
HC: Healthy control
MUC5AC: Mucin 5AC
PC: Pancreatic cancer
ROC: Receiver operating characteristics
